# Draft Genome Assembly of *Bordetella bronchiseptica* ATCC 10580, a Historical Canine Clinical Isolate

**DOI:** 10.1128/genomeA.00916-14

**Published:** 2014-09-18

**Authors:** H. E. Daligault, K. W. Davenport, T. D. Minogue, K. A. Bishop-Lilly, D. C. Bruce, P. S. Chain, S. R. Coyne, K. G. Frey, J. Jaissle, G. I. Koroleva, J. T. Ladner, C.-C. Lo, L. Meincke, C. Munk, G. F. Palacios, C. L. Redden, S. L. Johnson

**Affiliations:** a Los Alamos National Laboratory, Los Alamos, New Mexico, USA; b USAMRIID-DSD, Fort Detrick, Maryland, USA; c NMRC-Frederick, Fort Detrick, Maryland, USA; d Henry M. Jackson Foundation, Bethesda, Maryland, USA; e Center for Genome Sciences (CGS), United States Army Medical Research Institute of Infectious Diseases, Fort Detrick, Maryland, USA

## Abstract

We present the scaffolded genome of *Bordetella bronchiseptica* ATCC 10580, assembled into 98 contigs. This 5.1-Mb assembly (68.2% G+C content) contains 4,870 coding regions. The strain was originally isolated from canine lung tissue and is used in quality control testing.

## GENOME ANNOUNCEMENT

*Bordetella bronchiseptica* is a zoonotic pathogen causing canine tracheobronchitis, better known as “kennel cough” (1). The pathogen is commonly found in canines, felines, equines, and swine but is found only occasionally in humans (1–3). Recent studies suggest that *B. bronchiseptica* is ancestral to *Bordetella pertussis*, a human-specific pathogen that causes whooping cough (2, 4). Infection of humans by *B. bronchiseptica* appears limited to immunocompromised individuals (5, 6).

We sequenced the genome of *B. bronchiseptica* ATCC 10580, isolated prior to 1966 from canine lung tissue collected in Detroit, MI. The genome of this historical strain is provided to increase the number of reference genomes for diagnostic development and phylogenetic reconstructions.

High-quality genomic DNA was extracted from a purified isolate using the Qiagen Genomic-tip 500 at USAMRIID-DSD. Specifically, a 100-mL bacterial culture was grown to stationary phase and nucleic acid was extracted per the manufacturer’s recommendations. The draft genome sequence includes both Illumina and 454 data types. We constructed and sequenced a 100-bp Illumina library to 130-fold genome coverage as well as a separate long-insert paired-end library (6,648 ± 1,662-bp insert and 13-fold genome coverage) (Roche 454 Titanium platform) (7, 8). The two datasets were assembled together in Newbler (Roche) and the consensus sequences were computationally shredded into 2-kbp overlapping fake reads (shreds). Raw reads were also assembled in Velvet and those consensus sequences were computationally shredded into 1.5-kbp overlapping shreds (9). We then assembled all draft data using Allpaths and computationally shredded the consensus sequences into overlapping 10-kbp shreds (10). Finally, we used parallel Phrap (High Performance Software, LLC) to integrate the Newbler consensus shreds, Velvet consensus shreds, Allpaths consensus shreds, and a subset of the long-insert read pairs. Possible misassemblies were corrected and some gap closure was accomplished with manual editing in Consed (11–13).

Automatic annotation of the *B. bronchiseptica* ATCC 10580 genome utilized an Ergatis-based workflow at LANL with minor manual curation. The 5,133,086-bp genome (68.2% G+C content) includes 4,870 coding sequences (CDSs), 7 rRNAs, and 52 tRNAs in 98 contigs placed into a single scaffold. The annotated assembly has been deposited into NCBI and raw data files are available upon request.

### Nucleotide sequence accession number.

This genome has been deposited to GenBank under accession number JMRX00000000.
